# Gd^3+^–Trityl–Nitroxide Triple Labeling and Distance Measurements in the Heterooligomeric Cobalamin Transport Complex in the Native Lipid Bilayers

**DOI:** 10.1021/jacs.2c10080

**Published:** 2023-01-04

**Authors:** Sophie Ketter, Benesh Joseph

**Affiliations:** Institute of Biophysics, Department of Physics and Centre for Biomolecular Magnetic Resonance (BMRZ), Goethe University Frankfurt, Max-von-Laue-Str. 1, Frankfurt 60438, Germany

## Abstract

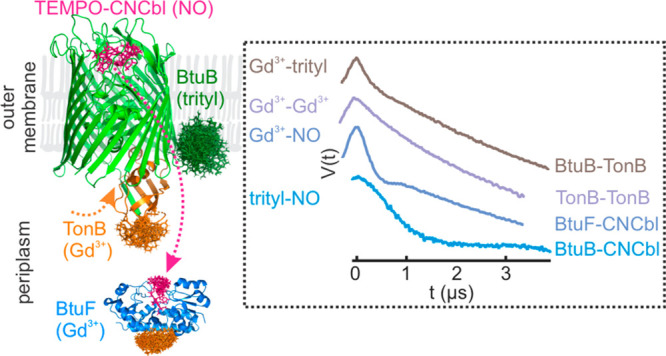

Increased efforts are being made for observing proteins in their native environments. Pulsed electron–electron double resonance spectroscopy (PELDOR, also known as DEER) is a powerful tool for this purpose. Conventionally, PELDOR employs an identical spin pair, which limits the output to a single distance for monomeric samples. Here, we show that the Gd^3+^–trityl–nitroxide (NO) three-spin system is a versatile tool to study heterooligomeric membrane protein complexes, even within their native membrane. This allowed for an independent determination of four different distances (Gd^3+^–trityl, Gd^3+^–NO, trityl–NO, and Gd^3+^–Gd^3+^) within the same sample. We demonstrate the feasibility of this approach by observing sequential ligand binding and the dynamics of complex formation in the cobalamin transport system involving four components (cobalamin, BtuB, TonB, and BtuF). Our results reveal that TonB binding alone is sufficient to release cobalamin from BtuB in the native asymmetric bilayers. This approach provides a potential tool for the structural and quantitative analysis of dynamic protein–protein interactions in oligomeric complexes, even within their native surroundings.

## Introduction

Observing the intermolecular interactions and their dynamics within a functional protein network calls for new approaches having high sensitivity and selectivity.^1−5^ Pulsed electron–electron double resonance spectroscopy (PELDOR or DEER) is the most popular tool to measure long-range distances in proteins, even within their native surroundings.^6−11^ The methane thiosulfonate nitroxide (NO) spin label (MTSL) combined with cysteine substitution is the most popular approach for labeling proteins.^12^ Other spin labels based on Gd^3+^, Mn^2+^, Cu^2+^, and trityl radicals are increasingly used, in particular for in situ studies.^13−18^ Typical PELDOR experiments employing an identical spin pair limit the output to a single distance. The presence of more than two identical spins as for the case of an oligomeric complex or proteins having multiple reactive cysteines would make data analysis and distance assignments very challenging.^19,20^ This motivated distance measurements between two different spin labels such as Gd^3+^–NO, Mn^2+^–NO, trityl–NO, Cu^2+^–NO, and Fe^3+^–NO.^21−30^ It was further extended into a Gd^3+^–Mn^2+^–NO three-spin system, which exhibits significant spectral overlap at W-band (95 GHz, ∼3.5 T).^31^

**Figure 1:**
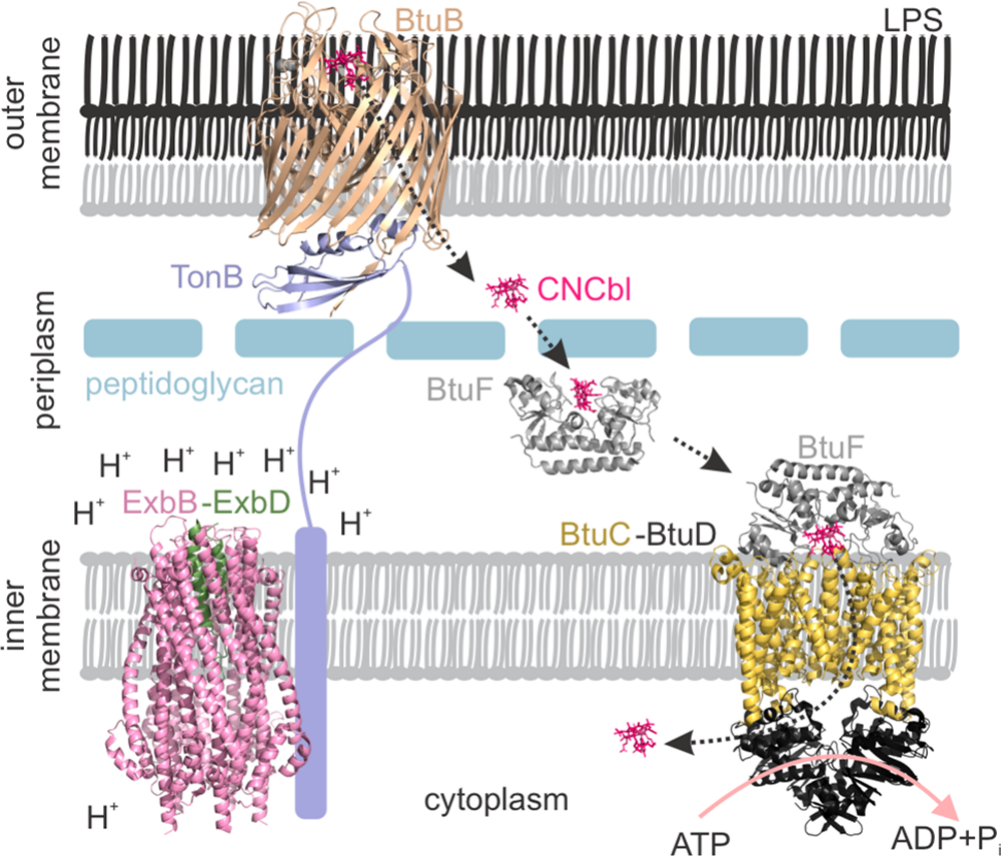
Cobalamin transport system in *E. coli*. BtuB–CNCbl–TonB_ΔTMD_ (PDB 2GSK), ExbB–ExbD (PDB 6TYI), BtuF–CNCbl (PDB 1N4A), and BtuCD–BtuF (PDB 2QI9) structures are shown.

**Figure 2:**
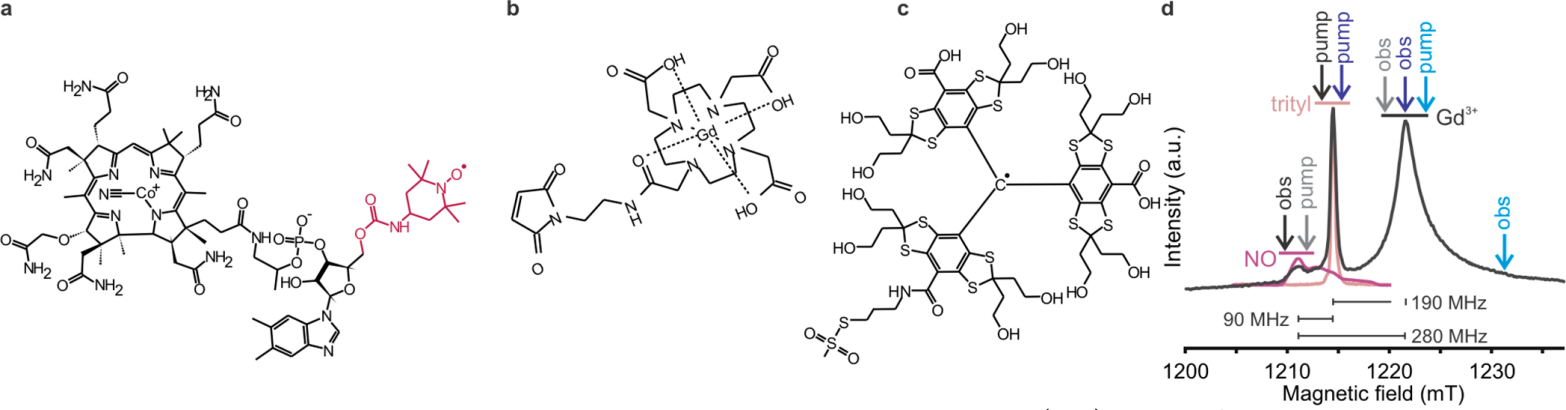
(a) The nitroxide labeled CNCbl analog (T-CNCbl), (b) M-Gd^3+^-DOTA, and (c) the MTS-OX063 trityl spin labels. (d) Echo-detected ESR (ED-ESR) spectrum of the T-CNCbl(NO)–BtuB(trityl)–TonB_ΔTMD_(Gd^3+^) complex in the native outer membranes. The NO and trityl spectra (vertically shifted and scaled) are overlaid to reveal the residual overlap. The offsets between the spectral maxima and the positions of the pump and observer pulses for trityl–NO (black), Gd^3+^–NO (gray), Gd^3+^–trityl (blue), and Gd^3+^–Gd^3+^ (cyan) PELDOR are indicated.

In this work, we present the first application of a Gd^3+^ (*S* = 7/2)–trityl (*S* = 1/2)–NO (*S* = 1/2) three-spin system, also for a membrane transport protein complex and in the native lipid bilayers (Figures 1 and 2). At the Q-band (34 GHz, ∼1.3 T), the central transition of Gd^3+^ (*M*_S_ = −1/2 to *M*_S_ = +1/2) is separated by ∼190 and ∼280 MHz from the maxima of the trityl and NO spectra, respectively (Figure 2d). Due to its short transversal relaxation time (*T*_2_), the Gd^3+^ signal is filtered while observing trityl or NO at ≥50 K. At lower temperatures, trityl and NO can be selectively excited by optimizing pulse lengths for a *S* = 1/2 system (a π pulse for trityl or NO equals 4π for the central transition of Gd^3+^). Trityl has a very narrow spectrum, and using appropriate pulses, it can be excited with minimal contribution from NO.

## Results and Discussion

### Spin Labeling the Components of the Cobalamin Transport Complex

Cyanocobalamin (CNCbl) transport in *E. coli* is achieved through a trans-envelope system spanning the inner membrane (IM), periplasm, and the outer membrane (OM) (Figure 1). The OM is an asymmetric bilayer consisting of phospholipids and lipopolysaccharides (LPSs). The effect of this asymmetry on the structure, dynamics, and function of the embedded proteins remains largely unknown. BtuB binds cobalamin from the extracellular space and transports it into the periplasm upon interaction with TonB, which transduces the energy from the ExbB–ExbD complex located in the IM. Five copies of ExbB and two copies of ExbD together form a proton channel and use the proton gradient across the IM.^32^ The energy derived from proton translocation is propagated to TonB, which interacts with the conserved Ton box sequence near the amino terminus of BtuB. In the periplasm, BtuF binds cobalamin and delivers it to the BtuCD complex located in the IM. The BtuCD–F complex finally transports cobalamin into the cytoplasm at the expense of ATP binding and hydrolysis. Despite the vast amount of the structural, biochemical, and biophysical data available, what triggers the CNCbl release from BtuB is still a matter of debate.^33−38^ Following the protocols previously established,^8,39,40^ here, we labeled BtuB T426C (using MTS-OX063 or MTSL) directly in the native OM. Briefly, after overexpression of BtuB in *E. coli*, cells were lysed and the total membrane fraction was separated. The IM was selectively solubilized using sarkosyl. The native outer membrane carrying BtuB was separated using ultracentrifugation, followed by spin labeling. TonB_ΔTMD_ I224C (without the single transmembrane helix) and the periplasmic cobalamin binding protein BtuF L232C were labeled with M-Gd^3+^-DOTA or MTSL as required (Figure S1; see Table S1 for labeling efficiencies). Finally, a TEMPO moiety was attached to CNCbl to create a labeled substrate analog (called T-CNCbl, Figure 2a).^39^

### PELDOR Spectroscopy Using Singly Labeled Components in the Native Membranes

**Figure 3:**
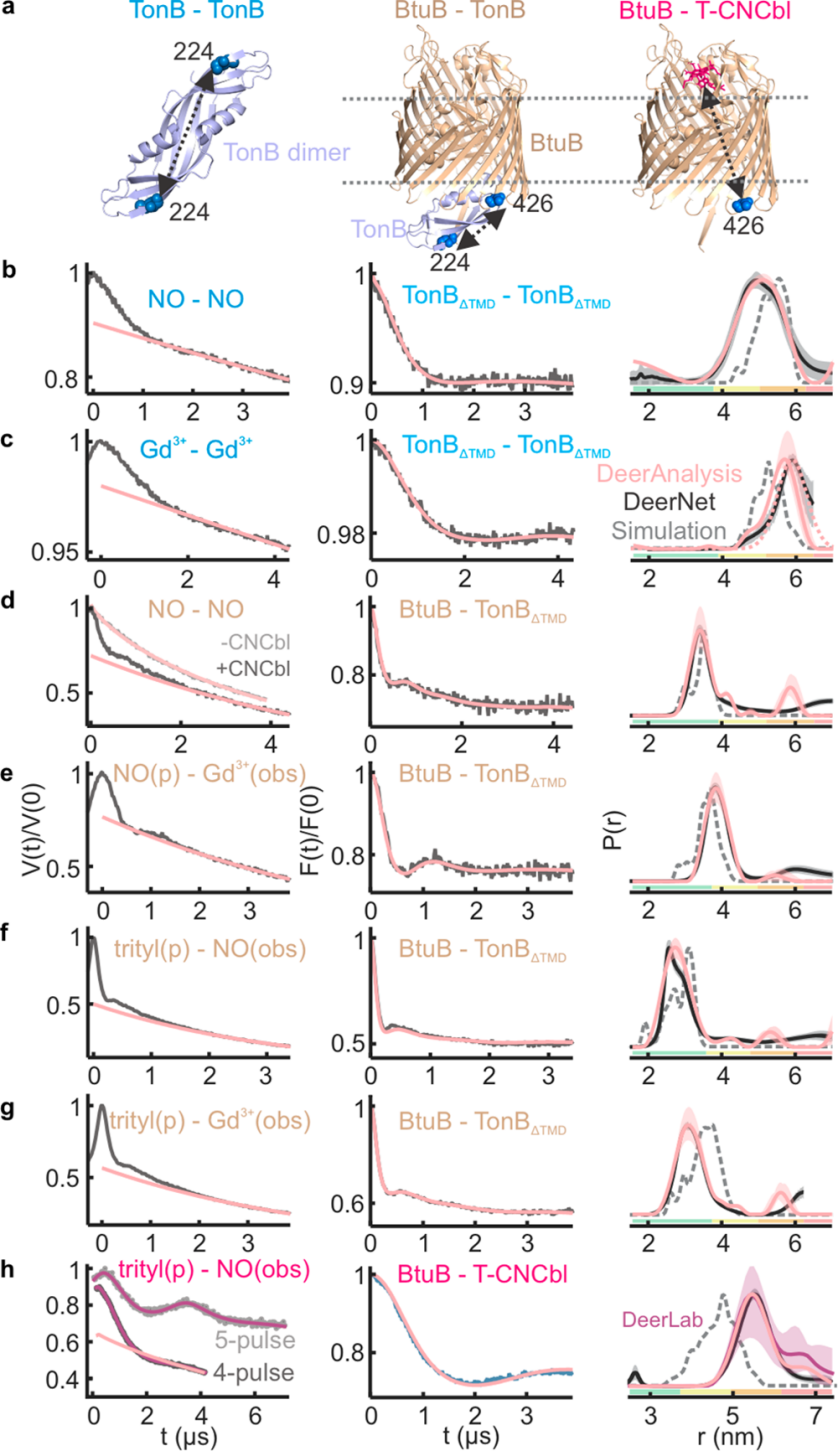
(a) The interactions observed in the native membrane are highlighted on the corresponding structures. (b–g) PELDOR data for TonB_ΔTMD_ dimers or BtuB–TonB_ΔTMD_ binding using NO, Gd^3+^, or OX063 trityl labels as indicated in the native membranes. (b, c) 36 μM TonB_ΔTMD_ was added to 18 μM BtuB, (d–g) 20 μM TonB_ΔTMD_ was added to 20 μM BtuB, or (h) 20 μM BtuB was mixed with 20 μM (4-pulse PELDOR) or 10 μM (5-pulse PELDOR) T-CNCbl. For P(r), distances obtained from Tikhonov regularization (TR) using DeerAnalysis^44^ and DEERNet^45^ are overlaid. The DeerLab^46^ analysis gave nearly identical outputs (Figure S5). For (c), the form factor corresponding to a single Gaussian fit (5.9 ± 0.4 nm, dotted pink line, see Figure S3) or (h) a two Gaussian fit (5.6 ± 0.4 and 6.8 ± 0.4 nm, pink line), respectively, is shown. In (h), the 4-pulse and 5-pulse PELDOR data were globally analyzed using the DeerLab software package. For the nonidentical spin pairs, the pump (p) and observer (obs) spins are indicated. Corresponding simulations (b and c using PDB 1IHR, d–g using PDB 2GSK, and h using PDB 1NQH) are overlaid (in gray dotted lines).

Initially, we characterized the interaction between different components employing two spin labels (Figure 3). Distance measurements using MTSL or Gd^3+^ labeled single cysteine variants showed that TonB_ΔTMD_ forms dimers in the presence of the native OM (Figures 3b,c and S2–S5 and Table S2). A smaller modulation depth (Δ, 10% vs ∼30% maximum for NO and 1.5% vs ∼4% maximum for Gd^3+^) suggests an equilibrium more favoring the monomers. The observed distance is in good agreement with the corresponding simulation on the structure of the dimeric C-terminal domain of TonB (PDB 1IHR). The simulations presented in this work were performed using a rotamer library for spin labeled cysteines as implemented in the MATLAB-based MMM program.^41^

The BtuB–TonB_ΔTMD_ interaction was observed using NO–NO, NO–Gd^3+^, trityl–NO, and trityl–Gd^3+^ PELDOR (Figure 3d–g with the first label always attached to BtuB). These extensive experiments revealed a narrow distance distribution in agreement with the simulations, thereby confirming that the larger size of the Gd^3+^ or trityl label does not cause any perturbation at the labeled sites. Knowing the labeling efficiency for TonB_ΔTMD_ with MTSL, these experiments enabled us to further estimate the degree of labeling with trityl (BtuB) and Gd^3+^ (TonB_ΔTMD_, Table S1).

In the presence of CNCbl (only), TonB_ΔTMD_ binds BtuB and the distances corresponding to the dimer were nearly absent (Figure 3d). Thus, CNCbl binding might expose the Ton box into the periplasm to which the TonB_ΔTMD_ monomer binds with a higher affinity, resulting in the dissociation of dimers.^33,42^ The cross-membrane distance between BtuB and T-CNCbl is beyond the range of the standard 4-pulse PELDOR. It was determined using the 5-pulse PELDOR sequence, which allows for the observation of a significantly longer dipolar evolution time window.^30,43^ This gave distances somewhat longer than the simulation (Figure 3h). Overall, the orthogonal pairs gave a larger Δ, especially while pumping trityl.

### Competitive Binding of T-CNCbl between BtuB and BtuF in the Native Membranes

**Figure 4:**
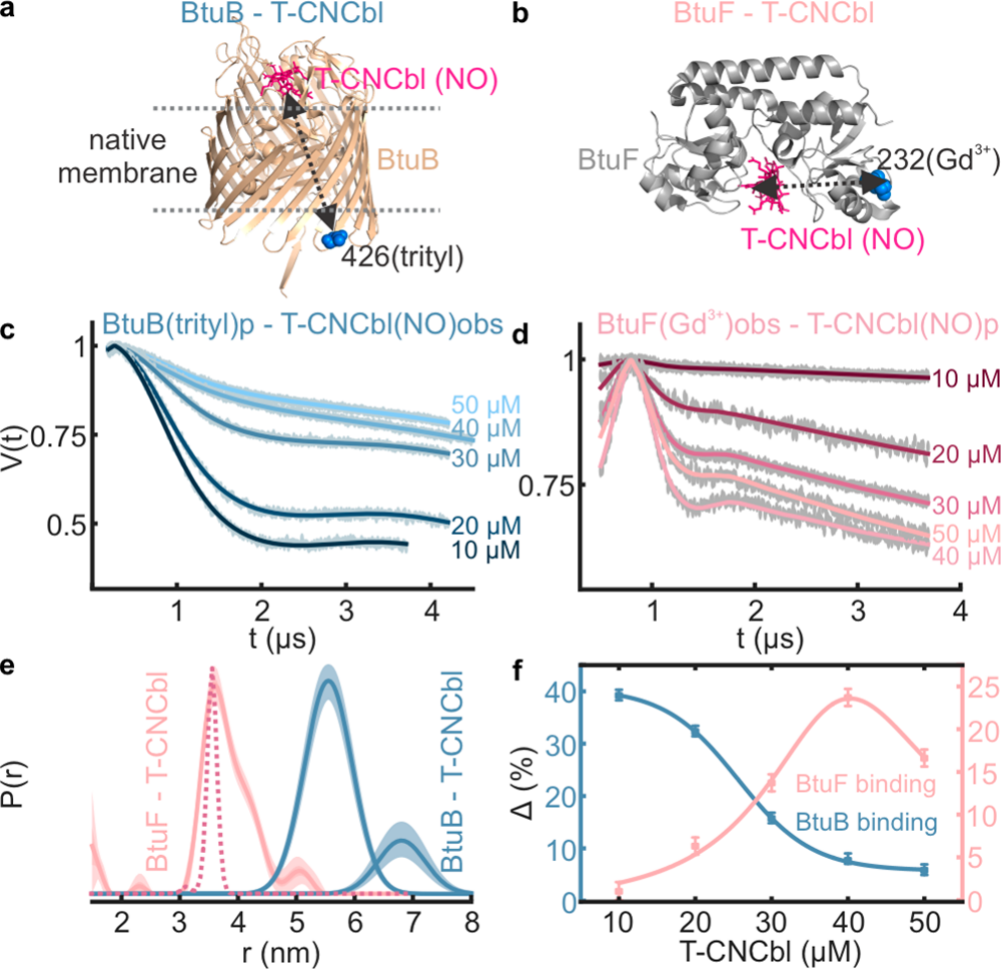
Selective trityl–NO and Gd^3+^–NO PELDOR of a mixture containing BtuB(trityl), BtuF(Gd^3+^), and T-CNCbl(NO) in the native membranes. 20 μM of BtuB and BtuF was mixed with the indicated concentrations of T-CNCbl. (a, b) The observed interactions are highlighted on the corresponding structures. The primary data were globally analyzed using (c) a two Gaussian model (Figure 2h) or TR (d) with the DeerLab program. (e) The obtained distance distributions and the simulation for BtuF–T-CNCbl distances (PDB 1N4A, dotted line) are shown. (f) The Δ values as obtained from (c) and (d) are plotted against T-CNCbl concentration. For BtuB and BtuF, saturation occurs at ≤10 and 40 μM, respectively. Excess T-CNCbl beyond these points further decreases the Δ.

Both BtuB and BtuF bind CNCbl with high (nM) affinity.^36^ We monitored the competitive binding of T-CNCbl analog using a three-spin system consisting of Gd^3+^ labeled BtuF and trityl labeled BtuB (Figure 4a,b). These data sets were globally analyzed to determine the distances as well as the extent of binding (Δ). BtuB–T-CNCbl (trityl–NO) data revealed a saturation binding (Δ_max_) already at the lowest concentration tested (10 μM), revealing a nM affinity (Figure 4c,f). Interestingly, BtuF–T-CNCbl binding (Gd^3+^–NO data) showed an opposite response with saturation close to 40 μM (Figure 4d,f). Thus, in the native environment, BtuB preferentially binds T-CNCbl and BtuF displays a significantly lower affinity (EC_50_ = 28 ± 1 μM). The TEMPO label on CNCbl projects out of the BtuF binding pocket, yet whether it has any role for the reduced affinity observed in the OM is unclear. These results clearly establish the potential of the three-spin system to simultaneously provide structural and quantitative information for competitive ligand/protein binding between different partners within a functional protein network.

### PELDOR Spectroscopy of the T-CNCbl–BtuB–TonB_ΔTMD_ Complex in the Native Membranes

**Figure 5:**
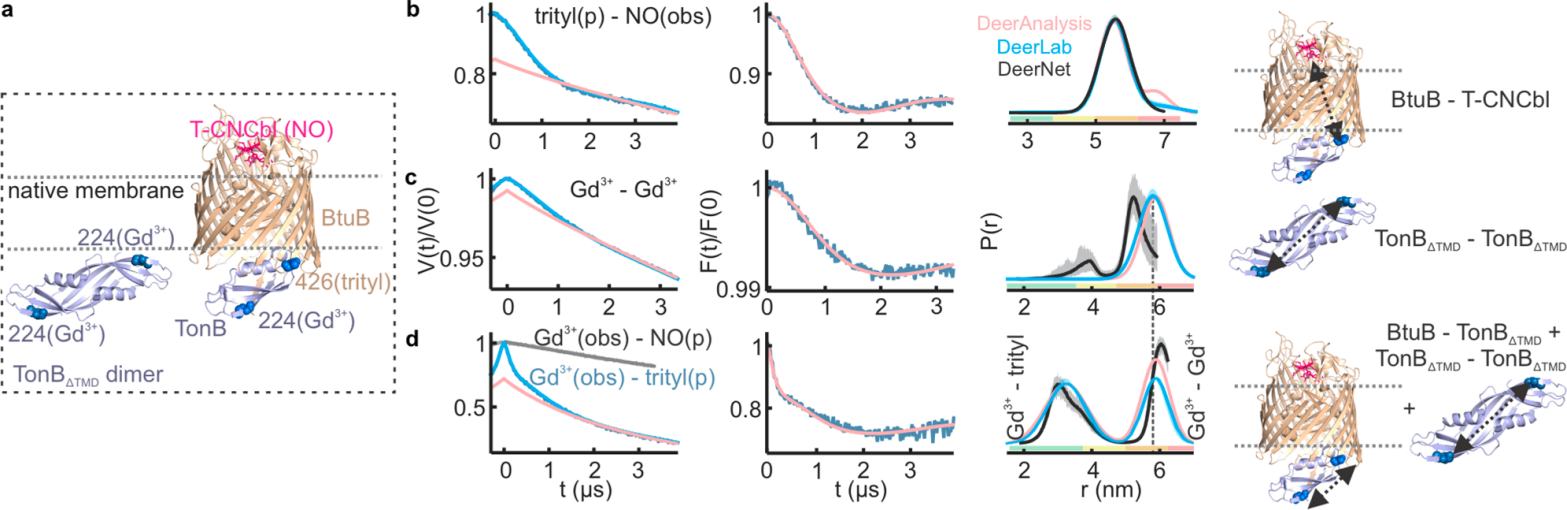
Selective trityl–NO, Gd^3+^–Gd^3+^, and Gd^3+^–trityl PELDOR on the T-CNCbl(NO)–BtuB(trityl)–TonB_ΔTMD (_Gd^3+^) complex in the native membranes. 20 μM each of BtuB, TonB_ΔTMD_, and T-CNCbl was mixed. (a) The sample consists of TonB_ΔTMD_ dimers and the T-CNCbl–BtuB–TonB_ΔTMD_ complex. The observed distances are highlighted on the corresponding structures in the rightmost panels. (b) The 4-pulse data was analyzed with the two Gaussian model (Figure 3h) using DeerLab and DeerAnalysis software packages. (c) Data is analyzed using a single Gaussian model (Figure 3c), and the difference for the DEERNet prediction is due to the limited observation time window. (d) Analysis was performed with a two Gaussian model corresponding to the Gd^3+^–trityl (3.2 ± 0.6 nm; see Figure 3g) and Gd^3+^–Gd^3+^ (see Figure 3c) distances; the latter appears as a crosstalk signal. Corresponding distances obtained using TR are shown in Figure S6. The Gd^3+^–NO PELDOR (in gray) did not reveal any distances. The DEERNet predictions are overlaid.

**Figure 6:**
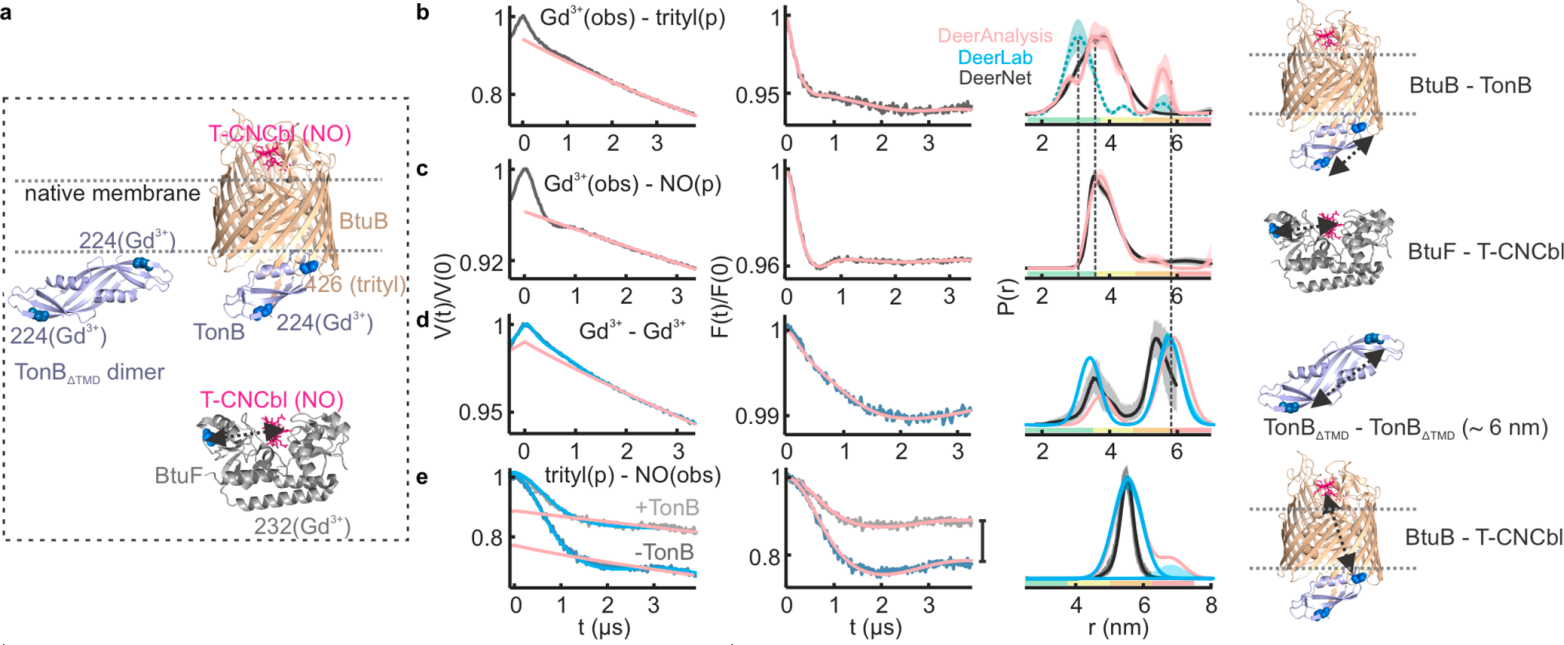
(a) Gd^3+^–trityl, Gd^3+^–NO, Gd^3+^–Gd^3+^, and trityl–NO PELDOR on the T-CNCbl(NO)–BtuB(trityl)–TonB_ΔTMD_(Gd^3+^) complex in the presence of BtuF(Gd^3+^) in the native membranes. 16 μM each of BtuB, TonB_ΔTMD_, and T-CNCbl was mixed with 23 μM T-CNCbl. The observed distances are highlighted on the corresponding structures in the rightmost panels. (b) The Gd^3+^–trityl PELDOR data and the distances obtained using TR. Corresponding distribution from Figure 3g is overlaid (see green), and the vertical lines indicate the *r*_max_ (see Figure S9 for DeerLab analysis). (c) Gd^3+^–NO PELDOR data and analysis. (d) The data is analyzed with a two Gaussian model corresponding to the Gd^3+^–Gd^3+^ distances for the TonB_ΔTMD_ dimer (see Figure 3c) and the first peak (3.7 ± 0.3 nm), which is well resolved from DEERNet and TR (Figure S8e). (e) The data were globally analyzed (DeerLab) using a two Gaussian function (see Figure 3h), and the difference in Δ is indicated with a vertical line. For (d, e), the output from TR is shown in Figure S8.

We reconstituted the T-CNCbl(NO)–BtuB(trityl)–TonB_ΔTMD_(Gd^3+^) complex in the native lipid bilayers (Figure 5a). At first, we detected BtuB–T-CNCbl binding using trityl-NO PELDOR. This gave distances identical with that observed in the absence of TonB_ΔTMD_. Yet, the Δ was significantly reduced (from 27 ± 4% to 16 ± 3%; see Figure 3h (4-pulse) vs Figure 5b), revealing that TonB_ΔTMD_ binding releases T-CNCbl from a fraction of BtuB (also see Figure 6). In line with this observation, earlier, we showed that the addition of TonB_ΔTMD_ increases the mobility of T-CNCbl to a level similar to the unbound form in the native OM.^36^ We next probed BtuB–TonB_ΔTMD_ binding using Gd^3+^–trityl PELDOR. Interestingly, this gave a bimodal distance distribution (Figure 5d). The first peak is identical with the Gd^3+^–trityl distance (Figure 3g), and the second peak corresponds to the Gd^3+^–Gd^3+^ distances observed for the TonB_ΔTMD_ dimer (Figure 3c).

For the Gd^3+^–NO PELDOR, it was shown that an additional crosstalk signal corresponding to the Gd^3+^–Gd^3+^ distance could appear due to the (suboptimal) coexcitation of underlying Gd^3+^ spins by the NO pump pulse.^47,48^ Their nutation experiments showed that, even at ∼290 MHz lower from the spectral maximum, pulses mostly excited the *M*_S_ = −1/2 to *M*_S_ = +1/2 transition. Pumping trityl could enhance this crosstalk signal due to the smaller frequency offset (Figure 2d). Another independent measurement further confirmed the presence of Gd^3+^–Gd^3+^ distances (Figure 5c). Evidently, the TonB_ΔTMD_ dimers did not completely dissociate in this sample (as opposed to Figure 3g). This could be due to an insufficient amount of BtuB and/or a surplus of TonB_ΔTMD_ within the error limits (±20%) or a somewhat inefficient unfolding of the Ton box induced upon the binding of the T-CNCbl analog. The simulation showed that Gd^3+^–NO (TonB_ΔTMD_–T-CNCbl) has a mean distance of ≥7 nm. This is beyond the limit of the observable dipolar evolution time, and PELDOR could not reveal any distances (Figure 5d, in gray on the left panel). Also, the partial release of T-CNCbl upon TonB_ΔTMD_ binding would significantly reduce the Δ in this case.

### PELDOR Spectroscopy of the T-CNCbl–BtuB–TonB_ΔTMD_ Complex in the Presence of BtuF in the Native Membranes

To further elucidate the role of BtuF in a more physiological context, we added Gd^3+^ labeled BtuF to the T-CNCbl(NO)–BtuB(trityl)–TonB_ΔTMD_(Gd^3+^) complex in the native membranes (Figure 6a). The presence of Gd^3+^ on both BtuF and TonB_ΔTMD_ would reduce Δ for the Gd^3+^–trityl and Gd^3+^–NO PELDOR, yet that does not hinder the determination of these distances. We performed four independent distance measurements on this sample (Figures 6 and S7–S9). For the Gd^3+^–trityl (TonB_ΔTMD_–BtuB) PELDOR (Figure 6b), we observed the presence of both Gd^3+^–NO and Gd^3+^–Gd^3+^ crosstalk signals (due to the coexcitation of Gd^3+^ and NO by the trityl pump pulse). The Gd^3+^–trityl peak gets broader and is shifted right by ∼0.5 nm (see green (from Figure 3g) vs pink lines). The amplitude of the Gd^3+^–Gd^3+^ crosstalk signal is decreased (DEERNet suggested a total absence; see Figure 6b vs 5d). This might be due to the presence of additional Gd^3+^ (BtuF) in the sample, which reduces the relative ratio between different spin pairs.^47^ The Gd^3+^–NO (BtuF–T-CNCbl) PELDOR revealed a somewhat longer distance (Figure 6c, also shown in Figure 4e), and a partial contribution (crosstalk) from it might account for the broadening of Gd^3+^–trityl distances. Importantly, no crosstalk signals (from Gd^3+^–Gd^3+^) were evident for the Gd^3+^–NO data under the experimental conditions. Purified BtuF and TonB_ΔTMD_ were shown to interact in solution.^49^ The Gd^3+^–Gd^3+^ PELDOR is intrinsically free from any crosstalk signals. Strikingly, the Gd^3+^–Gd^3+^ PELDOR revealed two peaks corresponding to the TonB_ΔTMD_ dimer and another peak at 3.7 ± 0.3 nm (Figure 6d). The latter peak could arise from the TonB_ΔTMD_–BtuF interaction. However, further experiments are necessary to rule out other possibilities (an alternate mode of TonB_ΔTMD_ dimerization or BtuF–BtuF interaction).

Finally, we determined the trityl–nitroxide (BtuB–T-CNCbl) distances in this sample (Figures 6e and S8). There was no visible contribution of any (Gd^3+^–NO) crosstalk signal in this case. However, as observed earlier (Figure 5b), the Δ was significantly reduced upon TonB_ΔTMD_ binding (from 22 ± 4% to 12 ± 2%), further confirming the release of T-CNCbl from a fraction of BtuB. Thus, under our experimental conditions, Gd^3+^–NO, Gd^3+^–Gd^3+^, and trityl–NO data are free of any crosstalk signals. The residual excitation of the underlying NO and Gd^3+^ spins while pumping trityl could generate Gd^3+^–NO and Gd^3+^–Gd^3+^ crosstalk signals into the Gd^3+^–trityl PELDOR data. We tested for any multispin effects while pumping trityl by employing pump pulses having varying inversion efficiencies,^20^ which did not reveal any visible contribution (data not shown). This is not unexpected considering the relatively low Δ and the only partial excitation of Gd^3+^ and NO spins by the pump pulse. When required, the relative contributions of the crosstalk signals could be further elucidated by optimizing the (trityl) pump pulse power for Gd^3+^, swapping the pump and observer positions, or changing the relative ratio between different spin pairs. A detailed characterization of these aspects for this three-spin system is beyond the scope of this work and awaits further investigation.

## Conclusions

In summary, our results establish the Gd^3+^–trityl–NO system as a versatile tool to study intermolecular interactions or competitive ligand binding in membrane protein complexes at the Q-band. This enabled the independent observation of four distances within the same sample. For an oligomeric complex or a protein–protein interaction network, it would be feasible to further increase the observable distances by introducing two copies of each label. However, depending on the labeling efficiencies and or the nature of the interactions, this can make the intersubunit distance distributions very broad and also lead to pronounced multispin effects and crosstalk signals. We show that TonB_ΔTMD_ exists in a dynamic monomer–dimer equilibrium, which shifts toward monomers upon interaction with the BtuB–CNCbl complex in the native membrane. TonB_ΔTMD_ binding is sufficient to release cobalamin from BtuB, which would be further taken by BtuF. Thus, an energy transduction by the ExbB–ExbD complex may be used to dissociate the BtuB–TonB interaction. Dynamic protein–protein interaction networks control molecular and cellular processes. The approach presented here offers a potential tool to elucidate the structural and dynamic basis of such complex processes, even in their native environments.

## Supporting Information Available

Protein expression, purification, membrane isolation, spin labeling, and continuous wave and pulsed ESR spectra and analysis (PDF)
