# Supplementary material for: Gd3+–Trityl–Nitroxide Triple Labeling and Distance Measurements in the Heterooligomeric Cobalamin Transport Complex in the Native Lipid Bilayers
Source: J Am Chem Soc. 2023 Jan 4;145(2):960–6. doi: 10.1021/jacs.2c10080 (PMC9853854; doi:10.1021/jacs.2c10080)
Supplement: Supplementary file 1 — ja2c10080_si_001.pdf [file ja2c10080_si_001.pdf]

Supporting Information

Gd^{3+} − trityl − nitroxide triple labeling and distance measurements in the heterooligomeric cobalamin transport complex in the native lipid bilayers

Sophie Ketter,[†] Benesh Joseph[†,*]

[†]Institute of Biophysics, Department of Physics and Centre for Biomolecular Magnetic Resonance (BMRZ), Goethe University Frankfurt, Max-von-Laue-Str. 1, 60438 Germany

*Email: joseph@biophysik.uni-frankfurt.de

Table of Contents

Experimental Procedures **3**

Mutagenesis and protein expression 3

Isolation of native outer membranes 3

Spin labeling and quantification of BtuB in the native outer membranes 3

Purification and spin labeling of $TonB_{\Delta TMD}$ 3

Purification and spin labeling of BtuF 4

SDS-PAGE analysis 4

Continuous-wave ESR spectroscopy 4

Pulsed ESR spectroscopy 4

Supplementary Figures **6**

Figure S1. Expression, purification, and spin labeling of BtuB T426C, $TonB_{\Delta TMD}$ I224C, and BtuF L232C 6

Figure S2. L-curves from Tikhonov regularization of PELDOR data for $TonB_{\Delta TMD}$ dimers or BtuB and $TonB_{\Delta TMD}$ binding 7

Figure S3. PELDOR data analysis (DeerAnalysis) for $TonB_{\Delta TMD}$ dimers 8

Figure S4. Deep neural network analysis for PELDOR measurements between $TonB_{\Delta TMD}$ – $TonB_{\Delta TMD}$ dimer, BtuB – $TonB_{\Delta TMD}$, and BtuB – T-CNCbl 9

Figure S5. DeerLab analysis of PELDOR data measured between $TonB_{\Delta TMD}$ – $TonB_{\Delta TMD}$ dimer and between BtuB and $TonB_{\Delta TMD}$ 10

Figure S6. Deep neural network analysis for PELDOR data measured on the BtuB – $TonB_{\Delta TMD}$ – T-CNCbl complex . 11

Figure S7. L-curves obtained from Tikhonov regularization of PELDOR data for the BtuB – $TonB_{\Delta TMD}$ – T-CNCbl complex in presence of BtuF 12

Figure S8. Deep neural network analysis for PELDOR data measured on the BtuB – $TonB_{\Delta TMD}$ – T-CNCbl complex in presence of BtuF 13

Figure S9. DeerLab analysis for PELDOR data measured between BtuB and $TonB_{\Delta TMD}$ in the BtuB – $TonB_{\Delta TMD}$ – T-CNCbl complex in presence of BtuF 14

Supplementary Tables **15**

Table S1. Spin labeling efficiencies for BtuB, $TonB_{\Delta TMD}$, and BtuF and the modulation depth values 15

Table S2. Error estimation for the four-pulse PELDOR measurements 16

References **17**

Experimental Procedures

Mutagenesis and protein expression

The cysteine variants of BtuB, $TonB_{\Delta TMD}$, and BtuF were created using the Q5 site-directed mutagenesis kit (E0554S, NEB) according to the protocol of the manufacturer. The pAG1 plasmid encoding the BtuB cysteine variant T426C was transformed into *E. coli* RK5016 (*metE70 argH btuB recA*). Cells were grown at 33°C in minimal medium consisting of 0.6 M K_2HPO_4, 0.33 M KH_2PO_4, 0.08 M $(NH_4)_2SO_4$ and 0.02 M sodium citrate supplemented with 0.01 % w/v L-arginine and L-methionine, 0.2 % w/v glucose, 150 µM thiamine, 3 mM $MgSO_4$, 300 µM $CaCl_2$, and 100 µg/mL ampicillin until an OD_{600} (optical density measured at a wavelength of 600 nm) value of 0.3 – 0.4. The cells were diluted 1:650 times into fresh minimal medium and grown overnight at 33°C until an OD_{600} of 0.4 – 0.5. The plasmid pHIS-parallel1 harboring the DNA fragment for $TonB_{\Delta TMD}$ (residues 33-239) from *E. coli* with an N-terminal 6xHis tag was used for protein expression.[1] The plasmid encoding the cysteine variant I224C was transformed into *E. coli* T7 Express lysY/I^q competent cells (C3013I, NEB). A single colony was used to inoculate 50 mL of LB medium supplemented with 100 µg/mL ampicillin and cells were grown overnight at 37°C. A 20 mL of the overnight culture was used to inoculate 2 L of 2xYT medium containing 100 µg/mL ampicillin, cells were cultured at 37°C and induced with 0.5 mM IPTG at an OD_{600} of 0.8. Post induction, cells were further grown at 20°C for 5 h. For expression of BtuF L232C, a construct containing an OmpA signal peptide sequence and a 6xHis tag fused to the C-terminal region was transformed into *E. coli* BL21-CodonPlus (DE3)-RIPL competent cells (230280, Agilent).[2] A 100 mL of TB medium supplemented with 100 µg/mL ampicillin was inoculated with a single colony and cells were grown overnight at 33°C. A 10 mL of the overnight culture was used to inoculate 1 L of TB medium containing 100 µg/mL ampicillin. The cells were cultured at 37 °C, induced with 0.4 mM IPTG at an OD_{600} of 0.7 and further grown at 25°C for 4 h. In all cases, cells were collected by centrifugation at 12,000x*g* and stored at – 80°C for further use.

Isolation of native outer membranes

To isolate outer membranes, *E. coli* RK5016 cells from a 2 L culture expressing BtuB were suspended in 50 mL of ice-cold 50 mM MOPS buffer (pH 7.5), 60 mM NaCl containing 10 µg/mL DNase I (DN25, Sigma-Aldrich) and one protease inhibitor cocktail tablet (4693159001, Roche) per 10 g of cell. Cells were lysed using a French press and the lysate was centrifuged at 15,000x*g* for 20 min at 4°C. The supernatant containing the total membrane was treated with 0.5 % N-lauroylsarcosine sodium salt for 5 min at room temperature to solubilize the inner membrane. The outer membrane fraction (~ 0.1 g) was collected by ultracentrifugation at 200,000x*g* for 1.5 h and suspended in 10 mL of 50 mM MOPS (pH 7.5), 60 mM NaCl.

Spin labeling and quantification of BtuB in the native outer membranes

Spin labeling of BtuB T426C was performed for 1 h at room temperature with 150 µM and 26 µM final spin label concentrations for 1-oxyl2,2,5,5-tetramethyl-3-pyrroline-3-methyl methanethiosulfonate (MTSL, Toronto Research Chemicals) and methanethiosulfonate functionalized OX063 (MTS-OX063),[3] respectively. The MTS-OX063 spin label was introduced into outer membrane vesicles by five cycles of freeze-thawing using liquid nitrogen. Excess spin label was removed by three rounds of centrifugation at 200,000x*g* for 1.5 h at 4°C and suspension in MOPS-NaCl buffer, which always included cycles of freeze-thawing for MTS-OX063. The outer membrane pellet was suspended in a final volume of ~ 100 µl using the same buffer. The outer membrane preparation was transferred to a micropipette for the continuous-wave (CW) ESR measurement. The amount of BtuB was assessed by a titration of TEMPO labeled cyano-cobalamin substrate (T-CNCbl) against the outer membrane preparation followed by a quantification of the bound fraction by CW ESR spectroscopy as we previously described.[4]

Purification and spin labeling of $TonB_{\Delta TMD}$

Purification was done adapting the protocols previously described.[1,5] To purify $TonB_{\Delta TMD}$, T7 Express lysY/I^q cells expressing cysteine variant I224C were suspended in ice-cold 25 mM Tris buffer (pH 7.5) containing 1 mM dithiothreitol (DTT), one protease inhibitor cocktail tablet (4693159001, Roche) per 10 g of cell, and 10 µg/mL of DNase I (DN25, Sigma-Aldrich). The cells were lysed by sonication and the cell lysate was pelleted by centrifugation at 73,000x*g* for 45 min at 4°C. The supernatant was stirred for 30 min at 4°C with 5 mL of Ni-NTA agarose resin (17-5268-01, Cytiva), which had been equilibrated in 25 mM Tris buffer (pH 7.5), 300 mM NaCl, 20 mM imidazole at 4°C. The mixture was loaded onto a PD-10 column (7322010, Bio-Rad) at room temperature followed by a first wash with three column volumes (CVs) of 25 mM Tris buffer (pH 7.5), 300 mM NaCl, 20 mM imidazole, and a second wash using two CVs of 25 mM Tris buffer (pH 7.5), 300 mM NaCl, 100 mM imidazole. The protein was eluted from the column with five CVs of 25 mM Tris buffer (pH 7.5), 300 mM NaCl, 250 mM imidazole. Fractions with the highest protein concentration were collected and imidazole was removed by a buffer exchange into 25 mM Tris buffer (pH 7.5), 300 mM NaCl. The eluate was diluted to 40 – 50 µM protein including 10% of glycerol and frozen in liquid nitrogen. Before spin labeling, the protein was treated with 5 mM of DTT for 15 min at room temperature and subsequently DTT was removed using a PD-10 column. For spin labeling with MTSL, a 25 mM Tris (pH 7.5), 300 mM NaCl buffer was used and the protein was

directly eluted from the PD-10 column into a falcon containing buffer to yield a final protein concentration of 7.5 μM and 40-fold MTSL. Spin labeling was done for 30 min at room temperature. In case of the maleimide functionalized M-Gd^{3+}-DOTA spin label, the pH of the buffer was adjusted to 6.8 and the protein was eluted from the column under argon atmosphere into a falcon containing buffer and 20-fold M-Gd^{3+}-DOTA which previously had been flushed with argon. Protein labeling took place overnight at 4°C for ~ 13 h under argon atmosphere. The protein was concentrated at 4°C and loaded onto a Superdex 200 Increase 10/300 GL column (17-5175-01, Cytiva), which had been equilibrated in 25 mM Tris (pH 6.8 or 7.5), 300 mM NaCl. Spin labeled $TonB_{\Delta TMD}$ was eluted and fractions corresponding to monomeric protein were combined. The protein was concentrated as described before to reach a final concentration of 80 – 110 μM. For MTSL labeled protein, the spin labeling efficiency was determined by CW ESR spectroscopy. Glycerol was added to a total concentration of 10% of and the protein was stored at – 80°C for further use.

Purification and spin labeling of BtuF

BtuF expression and purification were performed following protocols previously described.[2, 6] For the purification of BtuF, BL21-CodonPlus (DE3)-RIPL cells expressing variant L232C were suspended in ice-cold 25 mM Tris buffer (pH 7.5), 250 mM NaCl, 25 mM imidazole containing 5 mM β-Mercaptoethanol, one protease inhibitor cocktail tablet (4693159001, Roche) per 10 g of cell, 10 μg/mL of DNase I (DN25, Sigma-Aldrich), 50 μg/mL lysozyme (L6876, Sigma-Aldrich), and 10% of glycerol. Cells were lysed using sonication and the lysate was centrifuged at 73,000x*g* for 45 min at 4°C. All following steps were carried out at 4°C unless otherwise stated. The supernatant was incubated with 5 mL of Ni-NTA agarose resin (17-5268-01, Cytiva), which had been equilibrated in 25 mM Tris buffer (pH 7.5), 250 mM NaCl, 25 mM imidazole including 10% glycerol for 1 h and the mixture was loaded onto a PD-10 column (7322010, Bio-Rad). The column was washed with 10 CVs of 25 mM Tris buffer (pH 7.5), 250 mM NaCl, 25 mM imidazole with 10% glycerol followed by a second wash with 5 CVs of 25 mM Tris buffer (pH 7.5), 250 mM NaCl, 50 mM imidazole, 10% glycerol. Bound BtuF was eluted from the column with 5 CVs of 25 mM Tris buffer (pH 7.5), 250 mM NaCl, 300 mM imidazole, 10% glycerol. Fractions containing high yield of the protein were collected, diluted to 10 – 20 μM and treated with 5 mM DTT for 5 min. The protein was then buffer exchanged into 25 mM Tris buffer (pH 7.5), 300 mM NaCl, 10 % glycerol to remove imidazole and DTT using a PD-10 desalting column. The eluate from this column was directly collected into a falcon containing buffer to yield a final protein concentration of 4 – 5 μM and 20-fold M-Gd^{3+}-DOTA spin label which previously had been flushed with argon. Protein labeling was done overnight at 4 °C for ~13 h under argon atmosphere. The protein was then concentrated in a centrifugal filter unit with a 10 kDa molecular weight cutoff and loaded onto a Superdex 200 Increase 10/300 GL size-exclusion chromatography column (17-5175-01, Cytiva), which had been preequilibrated with 25 mM Tris (pH 6.8) containing 300 mM NaCl and 10% glycerol. Fractions corresponding to monomeric protein were combined and concentrated as described before to reach a final concentration of 90 – 150 μM. Subsequently, samples for ESR spectroscopy were prepared as described in the following sections.

SDS-PAGE analysis

Outer membrane and protein samples were mixed with 5x SDS loading dye (250 mM Tris HCl pH 8.0, 10% (w/v) SDS, 30% (v/v) glycerol, 0.05% (w/v) bromophenol blue, 100 mM dithiothreitol) and boiled at 99°C for 10 min. The samples were run on an 8 % polyacrylamide gel at 125 V. The gel was stained using coomassie dye (10% (w/v) Brillant blue R250, 5% (v/v) EtOH, 7.5% (v/v) HAc), destained and imaged for further analysis.

Continuous-wave ESR spectroscopy

All continuous-wave (CW) ESR spectra were recorded at X-band frequencies (9.4 GHz) as the first derivative on a Bruker EMXnano Benchtop Spectrometer operating at room temperature. The nitroxide spectra were acquired with 100 kHz modulation frequency, 0.15 mT modulation amplitude, 1.995 – 6.310 mW microwave power, 5.12 ms time constant, 22.5 ms conversion time and 18 mT sweep width. For samples containing trityl, the spectra were recorded with 100 kHz modulation frequency, 0.02 mT modulation amplitude, 1 mW microwave power, 1.28 ms time constant, 4.8 ms conversion time and 5 mT sweep width. For outer membrane samples, spectra were accumulated over 20 scans with a sweep time of 27 s and 12 s for nitroxide and trityl, respectively. Spectra of spin labeled $TonB_{\Delta TMD}$ were averaged over 20 scans. Quantification of the spin concentration of the samples was done using the reference-free concentration determination of paramagnetic species as implemented in the Bruker Xenon software.

Pulsed ESR spectroscopy

Spin labeled native outer membranes overexpressing BtuB were diluted to a final concentration of 20 μM of BtuB and TEMPO labeled cobalamin (T-CNCbl) was added in an equimolar concentration to BtuB. For experiments involving spin labeled $TonB_{\Delta TMD}$ in the outer membrane environment, 36 μM of protein was added to an unlabeled outer membrane preparation diluted to a final concentration of 18 μM of BtuB. $TonB_{\Delta TMD}$ was introduced into outer membrane vesicles by five cycles of freeze-thawing using liquid nitrogen. To perform pulsed measurements between $TonB_{\Delta TMD}$ and BtuB in isolated native outer membranes, the outer membranes were diluted to a final concentration of 20 μM of BtuB and $TonB_{\Delta TMD}$ was added in an equal molar concentration to BtuB in the presence of 1 mM $CaCl_2$ and 33 – 100 μM cobalamin

followed by five cycles of freeze-thawing. Additionally, a sample was prepared in the absence of cobalamin. For the titration experiment of BtuB in native outer membranes and BtuF against T-CNCbl, samples were prepared with final protein concentrations of 20 µM, 1 mM $CaCl_2$ and the ligand concentration was varied from 10 – 50 µM. To prepare the BtuB – $TonB_{\Delta TMD}$ – T-CNCbl complex, $TonB_{\Delta TMD}$ and T-CNCbl were added to the native outer membranes containing BtuB to reach a final concentration of 20 µM for each molecule. Similarly, for the BtuB, $TonB_{\Delta TMD}$, BtuF, and T-CNCbl sample, the components were mixed to reach a final concentration of 16 µM for the proteins and 23 µM for T-CNCbl. The sample was freeze-thawed as described before. A 20% d_8-glyerol was added to all samples, subsequently transferred to 1.6 mm outer diameter quartz ESR tubes (Suprasil, Wilmad LabGlass) and snap-frozen in liquid nitrogen.

PELDOR/DEER measurements were performed on a Bruker Elexsys E580 Q-Band Pulsed spectrometer equipped with an arbitrary waveform generator (SpinJet-AWG), a continuous-flow helium cryostat, a temperature control system (Oxford Instruments), a 50 W solid state amplifier, and a Bruker EN5107D2 dielectric resonator. Echo-detected ESR spectrum and T_M measurements were performed at 10 K for Gd^{3+} or at 50 K for nitroxide and trityl using the two pulse Hahn echo sequence $\pi/2 - \tau - \pi - \tau$ employing Gaussian pulses (32 ns or 48 ns) and an inter-pulse delay (τ) of 400 ns. The values for T_M were obtained by a fit of a stretched exponential function $\exp[-(2\tau/T_M)^{\kappa}]$ to the baseline corrected and normalized data. The four-pulse PELDOR experiments were recorded using a dead-time free sequence employing Gaussian pump and observer pulses and a 16-step phase cycling (x[x][xp]x).[7-8] Nitroxide – nitroxide measurements were acquired at 50 K using a 38 – 40 ns pump pulse and 48 ns observer pulses. The pump pulse was set to the maximum of the echo-detected field-swept spectrum, while the observer pulses were set at 80 MHz lower and the shot repetition time (SRT) was kept at 2 ms. For nitroxide – trityl PELDOR, the temperature was set to 50 K and a 48 ns pump pulse was applied at the maximum of the trityl spectrum. The 48 ns observer pulses were set 90 MHz higher and a SRT of 2 ms was used. Nitroxide – Gd^{3+} PELDOR experiments were recorded at 10 K while pumping the nitroxide and observing the Gd^{3+}. The pump pulse length was always set to 24 ns, while 32 or 48 ns observer pulses (depending on the available power) were set at 280 MHz lower. The SRT was kept at 1 ms. Trityl – Gd^{3+} measurements were performed at 10 K, while pumping at the maximum of the trityl spectrum and observing the Gd^{3+} with a frequency offset of 190 MHz. The pump pulse was always set to 38 ns and the observer pulses were kept at 32 ns with a SRT of 1 ms. To perform Gd^{3+} – Gd^{3+} experiments, the temperature was set to 10 K with a SRT of 1 ms. A 32 ns pump pulse was placed to the maximum of the Gd^{3+} spectrum, while the 32 ns observer pulses were applied at 100 MHz lower. The deuterium modulations were averaged by increasing the first inter pulse delay by 16 ns for 8 steps for all samples. The nitroxide – trityl five-pulse PELDOR experiment was recorded at 50 K using 16-step phase cycling (x[x][xp]x) and Gaussian pump and observer pulses. The pump pulses were set to the maximum of the trityl spectrum and the 48 ns observer pulses were set at 90 MHz lower to the maximum of the nitroxide spectrum and a SRT of 2 ms was used. The length of the first static pump pulse and the second time-varied pump pulse was kept at 38 ns and 64 ns, respectively. The deuterium modulations were averaged by increasing the first inter pulse delay by 16 ns for 8 steps and correspondingly shifting the static pump pulse.

Distance distributions were independently determined using DeerAnalysis,[9] DeerLab[10] and the DEERNet[11] programs. For DeerAnalysis, background function arising from intermolecular interactions was subtracted from the normalized primary PELDOR data V(t)/V(0). The resulting form factors F(t)/F(0) were fitted to distance distributions using a model-free Tikhonov regularization (TR) or a Gaussian model-based approach as described. The suggested regularization parameter (α) for TR was chosen according to the L-curve corner recognition as implemented in the DeerAnalysis software. To estimate the uncertainty for the probability distribution, distances for different background functions were determined by a variation of the time window and the dimensionality for the spin distribution (**see Table S2**). For DeerLab, the primary data were simultaneously fitted for the distances and the background function using a model-free TR or a Gaussian model-based approach as indicated. Finally, the data were analyzed to predict the distances (and the background) in a user-independent manner using the deep neural network (DEERNet)[11,12], as featured in the DeerAnalysis software packages. For related data sets (as in Figures 3h and 4c-d), a global analysis was performed using DeerLab. Trityl-nitroxide 5-pulse PELDOR was performed as we previously described.[13]

Simulations of the distance distributions were obtained on the structures of calcium and cyanocobalamin bound BtuB (PDB 1NQH), BtuB – cobalamin – TonB complex (PDB 2GSK), cyanocobalamin bound BtuF (PDB 1N4A), and the dimeric C-terminal domain of TonB (PDB 1IHR) using the rotamer libraries for MTSL, MTS-OX063, and DOTA-Gd as implemented in the MATLAB-based software package MMM2022.2.[12]

Supplementary Figures

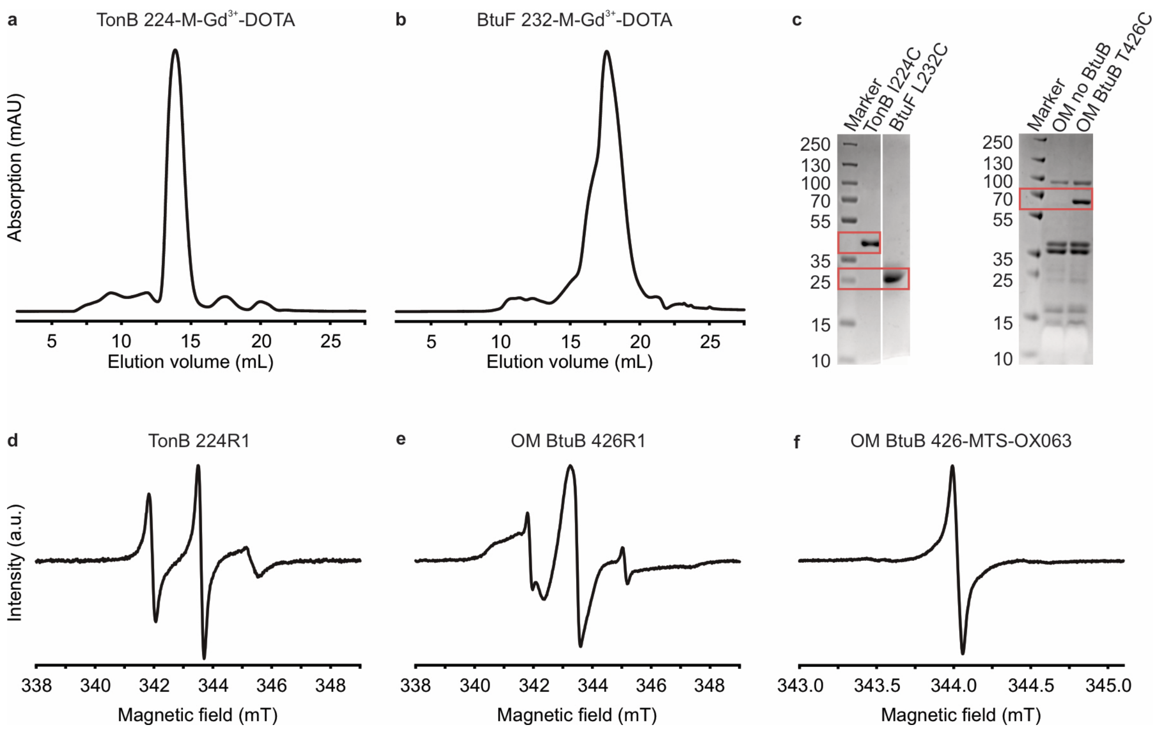

Figure S1. Expression, purification, and spin labeling of BtuB T426C, $TonB_{\Delta TMD}$ I224C, and BtuF L232C. (a, b) Elution profiles obtained from size-exclusion chromatography (SEC) for spin labeled $TonB_{\Delta TMD}$ and BtuF as indicated in the figure. (c) On the left, SDS-PAGE showing the bands corresponding to spin labeled $TonB_{\Delta TMD}$ and BtuF (~ 26 kDa and ~ 30 kDa, respectively, highlighted inside the red box) obtained from SEC, which were used to perform further ESR experiments. $TonB_{\Delta TMD}$ migrated to a higher molecular weight of > 35 kDa, which is attributed to its polyproline motif.[14] On the right, SDS-PAGE showing the expression of BtuB in the isolated native outer membranes (~ 68 kDa). The band corresponding to BtuB was confirmed by isolation of outer membranes from cells grown in the absence of the plasmid (indicated as OM no BtuB). (d-f) Room temperature continuous wave (CW) ESR spectra of MTSL labeled $TonB_{\Delta TMD}$ I224C and of MTSL or MTS-OX063 labeled BtuB T426C in the isolated native outer membranes. The spin concentration obtained from the spectrum for $TonB_{\Delta TMD}$ 224R1 was used to calculate the MTSL spin labeling efficiency and amounted to ~ 94%. BtuB in our outer membrane preparations was quantified using the TEMPO modified cobalamin substrate (T-CNCbl) and was found to be ~ 20 μM. However, labeling of BtuB in these membranes using MTSL and MTS-OX063 gave a larger spin concentration (~ 100 μM and ~ 80 μM, respectively) due to non-specific binding (OM proteins are devoid of any reactive cysteines). This does not lead to any distances, other than somewhat decreasing the overall modulation depth.[14-17]

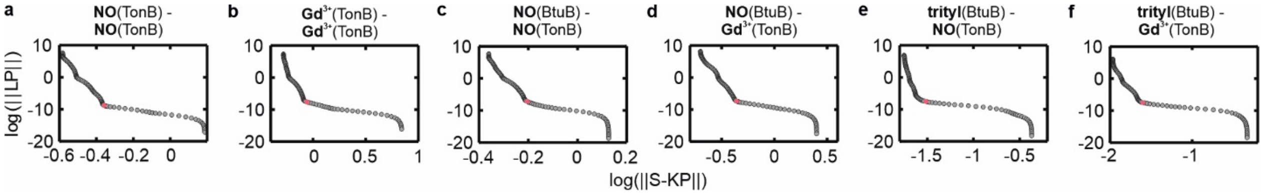

Figure S2. (a-f) L-curves obtained from the Tikhonov regularization (DeerAnalysis) of PELDOR data for $TonB_{\Delta TMD}$ dimers or BtuB and $TonB_{\Delta TMD}$ binding as shown in Figure 3. The regularization parameter used is highlighted in pink.

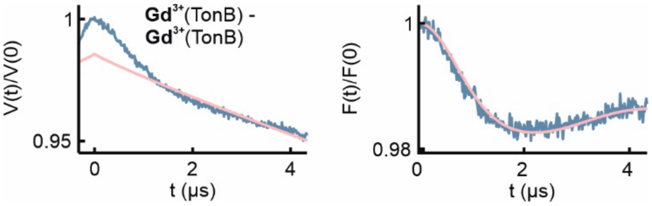

Figure S3. Tikhonov regularization analysis (DeerAnalysis) of PELDOR data for $TonB_{\Delta TMD}$ dimers in the native membrane environment using a single Gaussian fitting as presented in Figure 3c. Left panel shows the primary Gd^{3+}-Gd^{3+} PELDOR data (in blue) with the intermolecular (background) function overlaid (in pink). Right panel, the background corrected form factor with the corresponding fit overlaid.

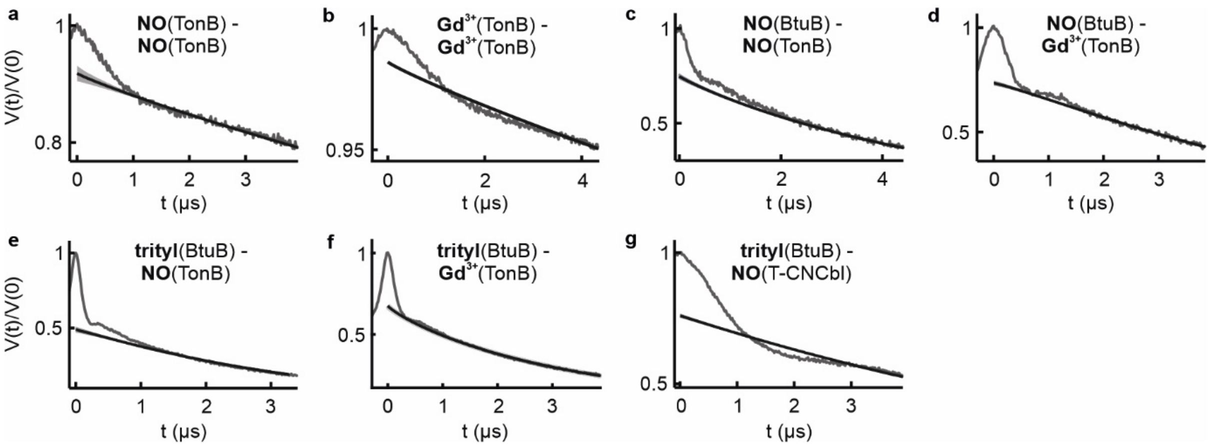

Figure S4. (a-g) Deep neural network analysis (DeerNet) of PELDOR data between $TonB_{\Delta TMD}$ - $TonB_{\Delta TMD}$ dimer, BtuB and $TonB_{\Delta TMD}$, and BtuB and the spin labeled substrate (T-CNCbl). The corresponding distance distributions are presented in Figure 3. The predicted intermolecular (background) function (in black) is overlaid with the primary PELDOR data (in light grey).

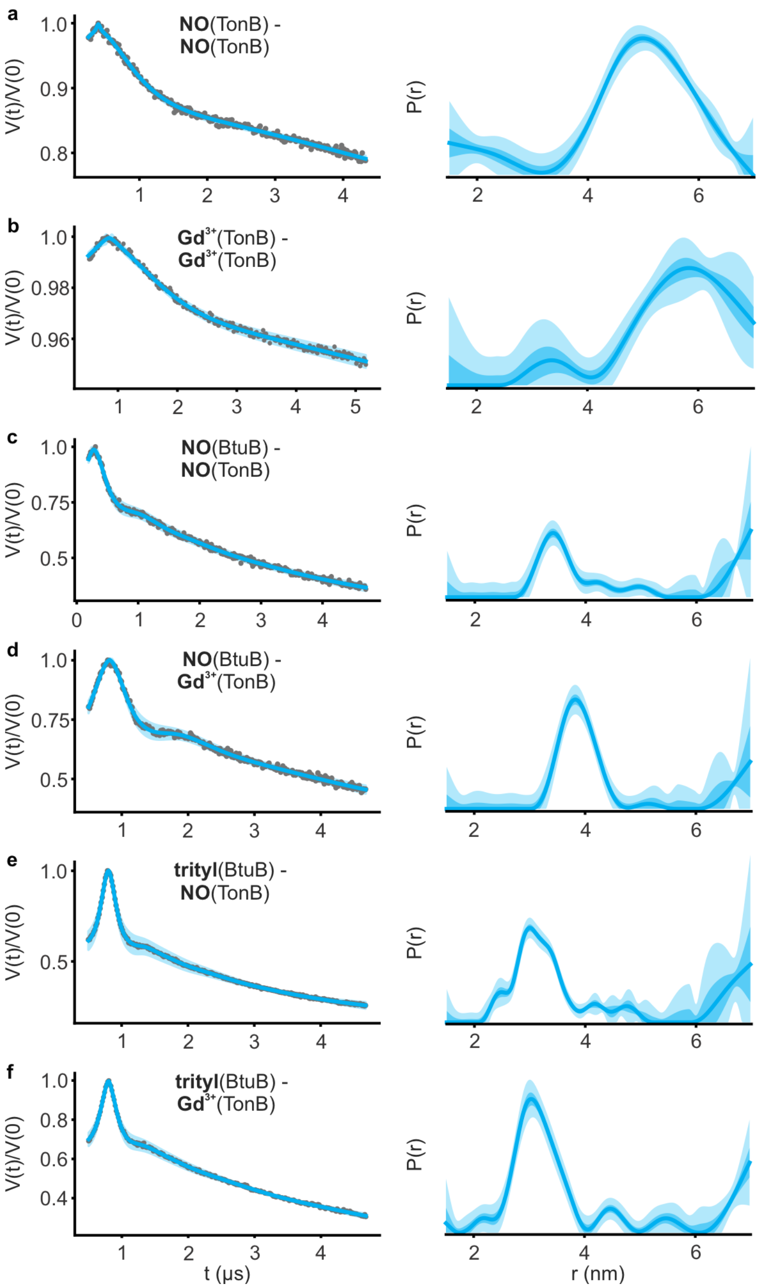

Figure S5. Tikhonov regularization analysis (DeerLab) of PELDOR data measured between $TonB_{\Delta TMD}$ - $TonB_{\Delta TMD}$ dimer and BtuB and $TonB_{\Delta TMD}$ (see Figure 3 for corresponding DeerAnalysis and DeerNet results). (a-f) Left panels, the primary PELDOR data (in light grey) overlaid with the corresponding fit (in blue). The right panels show the resulting distance distributions. Overall, the distances are nearly identical to the results obtained using DeerAnalysis and DEERNet (Figure 3b-g).

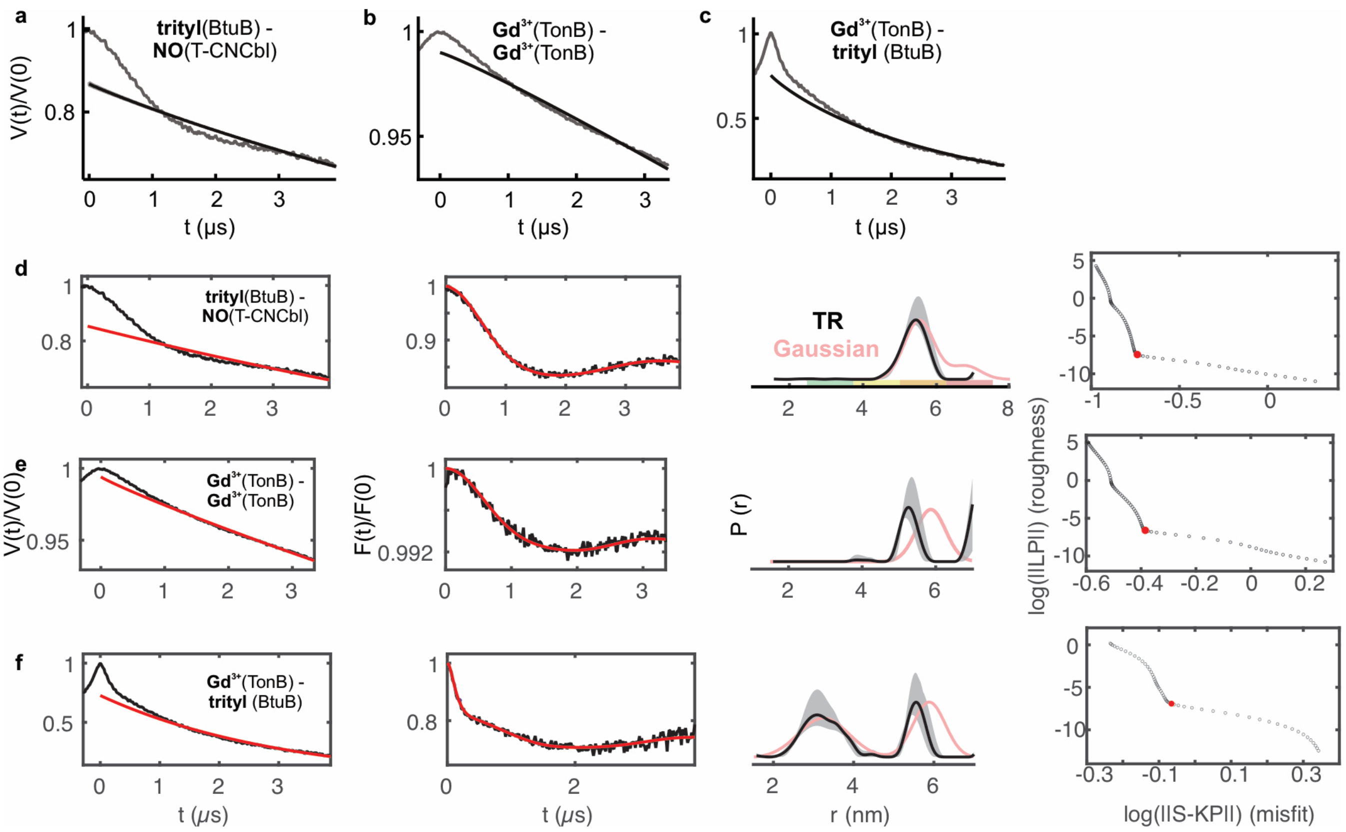

Figure S6. (a-c) Deep neural network analysis (DEERNet) for PELDOR data measured on the BtuB – $TonB_{\Delta TMD}$ – T-CNCbl complex in the native outer membranes as indicated in the figure. The corresponding distance distributions are presented in Figure 5. The primary PELDOR data (in light grey) is overlaid with the predicted intermolecular (background) function (in black). (d-f). Analysis of the above datasets employing Tikhonov regularization (TR) using DeerAnalysis. The primary data overlaid with the background function, form factor, the obtained distance distribution and the L-curves are shown. The uncertainty for the distance distribution was estimated by changing the length of the background function (as suggested by the program in 11 steps) and the dimensionality for the background function (d = 2.5 – 3.0 in 5 steps). Distance distribution obtained with a Gaussian model (which is obtained from independent experiments, see Figure 3) based analysis (as shown in Figure 5) is overlaid (in pink) for each sample. Overall, due to the limited dipolar evolution window which could be observed in the native membrane environment, for longer distances (> 4 nm) TR gave somewhat smaller distances, particular for Gd^{3+}-Gd^{3+} experiment.

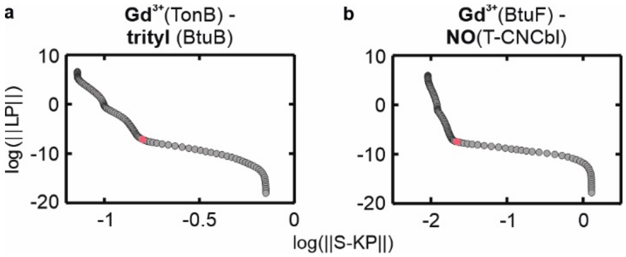

Figure S7. (a-b) L-curves obtained from Tikhonov regularization (DeerAnalysis) of PELDOR data measured on the BtuB – $TonB_{\Delta TMD}$ – T-CNCbl complex in presence of BtuF in the native outer membranes. The corresponding data and results are shown in Figure 6b-c. The regularization parameter used is highlighted in pink.

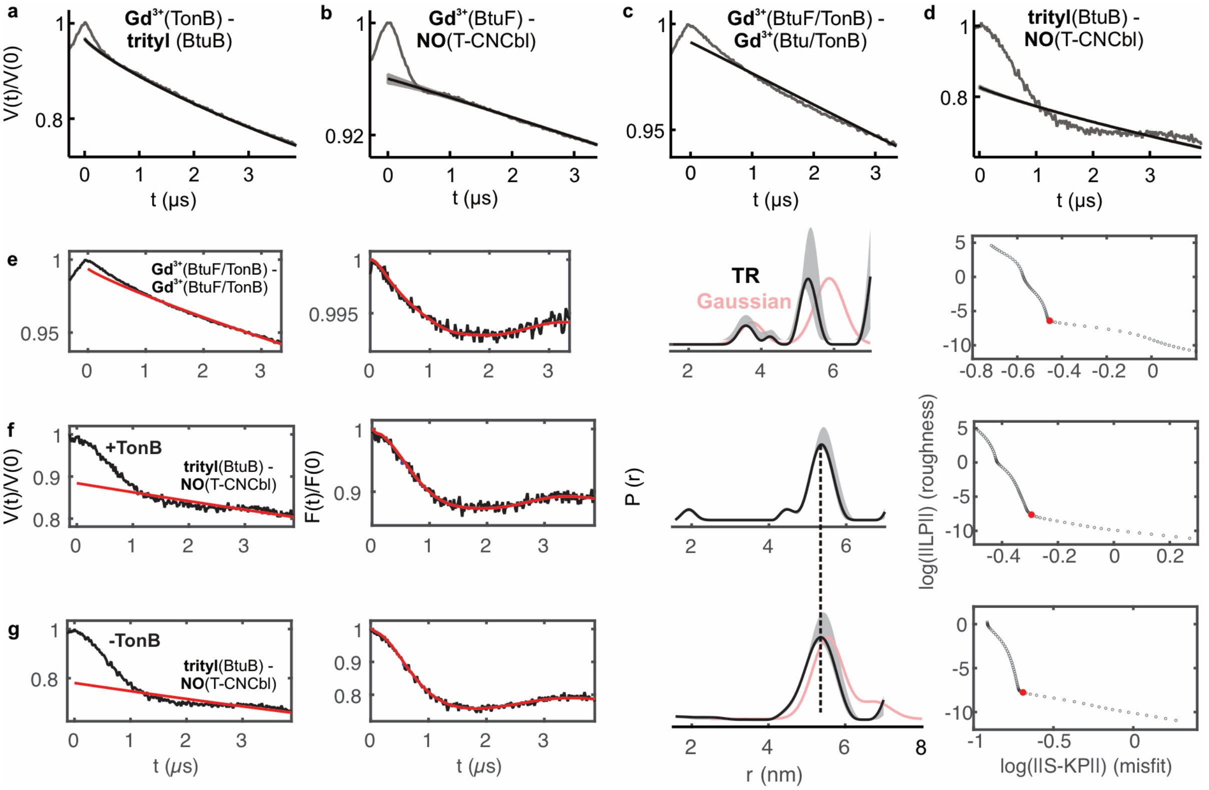

Figure S8. (a-d) Deep neural network analysis (DEERNet) for PELDOR data measured on the BtuB – $TonB_{\Delta TMD}$ – T-CNCbl complex in presence of BtuF in the native outer membranes as indicated. The corresponding distance distributions are presented in Figure 6. The primary PELDOR data (shown in light grey) is overlaid with the predicted intermolecular (background) function (black). (e-g) Analysis of the indicated datasets employing Tikhonov regularization (TR) using DeerAnalysis. The primary data overlaid with the background function, form factor, the obtained distance distribution and the L-curves are shown. The uncertainty for the distance distribution was estimated by changing the length of the background function (as suggested by the program in 11 steps) and the dimensionality for the background function (d = 2.5 – 3.0 in 5 steps). Distance distribution obtained with a Gaussian model (which is obtained from independent experiments) based analysis (as shown in Figure 6) is overlaid (in pink) for each sample. Overall, due to the limited dipolar evolution window, which could be observed in the native membrane environment, for longer distances (> 4 nm) TR gave somewhat smaller distances, in particular for the Gd^{3+}-Gd^{3+} experiment.

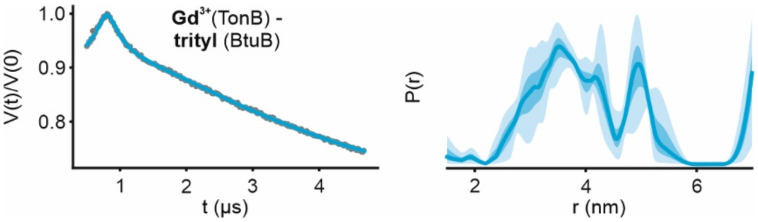

Figure S9. DeerLab analysis for PELDOR data measured between BtuB and TonB$_{\Delta TMD}$ in the BtuB – TonB$_{\Delta TMD}$ – T-CNCbl complex in presence of BtuF in the native outer membranes. Left panel shows the primary PELDOR data (in light grey) with the corresponding fit overlaid (in blue). The right panel shows the resulting distance distribution. The DeerAnalysis and DEER-Net outputs are shown in Figure 6b.

Supplementary Tables

Table S1. Spin labeling efficiencies (L.E.) for BtuB (in the native outer membranes), $TonB_{\Delta TMD}$, and BtuF with MTSL, M-Gd^{3+}-DOTA, or MTS-OX063 as indicated. The labeling efficiency for $TonB_{\Delta TMD}$ with MTSL amounted to ~ 94% as quantified using CW ESR spectroscopy (Figure S1). The labeling efficiency for the TEMPO modified substrate (T-CNCbl) is estimated to be ~ 70%.[4] The labeling efficiency for the other cases can be estimated from the modulation depth parameter, which is defined as [inversion efficiency of the pump pulse × labeling efficiency of the protein]. Under our experimental conditions and when pumping nitroxide, the inversion efficiency of the pump pulse is ~ 30% yielding a Δ of ~ 30% for a 100% labeling case. When pumping the narrow trityl spectrum, an inversion efficiency of ~ 100% could be achieved resulting in a Δ of ~ 100% for a 100% labeling case. Briefly, the L.E. of OX063 trityl or MTSL for BtuB in the membranes was estimated from the trityl(BtuB) – NO($TonB_{\Delta TMD}$) or NO(BtuB) – NO($TonB_{\Delta TMD}$) PELDOR respectively. This further helped to estimate the L.E. of M-Gd^{3+}-DOTA for $TonB_{\Delta TMD}$ from the NO(BtuB) – Gd^{3+}($TonB_{\Delta TMD}$) or trityl(BtuB) – Gd^{3+}($TonB_{\Delta TMD}$) PELDOR. The L.E. values are estimated to have a 10-15 % error.

Sample	Figure	Δ (%)	L.E. (%)
TonB(NO)			94
T-CNCbl			70
Trityl(BtuB) – NO(TonB)	3f	47.6	BtuB(trityl)<br>51
NO(BtuB) – NO(TonB)	3d	23.8	BtuB(NO)<br>84
NO(BtuB) – Gd^{3+}(TonB)	3e	23.7	TonB(Gd^{3+})<br>90
Trityl(BtuB) – Gd^{3+}(TonB)	3g, 5d, 6b	34.6 (2f)	70
Gd^{3+}(BtuF) – NO(T-CNCbl)	3b	23.7	BtuF(Gd^{3+})<br>100

Table S2. Error estimation for the four-pulse PELDOR measurements corresponding to the data presented in Figure 3 and 6 as indicated in the table. The data was analyzed using two-step Tikhonov regularization as implemented in the DeerAnalysis2021b software package. Probability distributions were calculated for different background models from a combined variation of the starting time (in the indicated range in 11 steps) and the dimensionality of the background function (d = 2.5 – 3 in steps of 5 unless otherwise stated). The value $\boldsymbol{t_{max}}$ indicates the maximum dipolar evolution time observed for the primary data. The resulting uncertainty bands are presented with the distance distributions. A prune level $\boldsymbol{L_{\mathrm{prune}}}$ of 1.15 of the root mean square deviation (r.m.s.d.) of the best fit was used for error estimation.

Sample	Figure	Error validation					Regularization parameter (α)
		Dimensionality		Starting time window			
		Range	Steps	Range (ns)	Steps	t_{max} (µs)	
**TonB**							
NO(TonB) – NO(TonB)	3b	2.7-3	3	784-2368	11	3.9	794
Gd^{3+}(TonB) – Gd^{3+}(TonB)	3c	2.7-3	3	864-2608	11	4.4	794
**BtuB - TonB**							
NO(BtuB) – NO(TonB)	3d	2.5-3	5	880-2656	11	4.4	158
NO(BtuB) – Gd^{3+}(TonB)	3e	2.5-3	5	768-2320	11	3.9	251
Trityl(BtuB) – NO(TonB)	3f	2.5-3	5	688-2048	11	3.3	158
Trityl(BtuB) – Gd^{3+}(TonB)	3g	2.5-3	5	768-2320	11	3.9	126
**BtuB – TonB – BtuF – T-CNCbl**							
Gd^{3+}(TonB) – trityl(BtuB)	6b	2.5-3	5	768-2320	11	3.9	126
Gd^{3+}(BtuF) – NO(T-CNCbl)	6c	2.5-3	5	672-2032	11	3.4	158

References

1. Freed, D. M.; Lukasik, S. M.; Sikora, A.; Mokdad, A.; Cafiso, D. S., Monomeric TonB and the Ton box are required for the formation of a high-affinity transporter-TonB complex. *Biochemistry* **2013**, *52* (15), 2638-2648.
2. Hvorup, R. N.; Goetz, B. A.; Niederer, M.; Hollenstein, K.; Perozo, E.; Locher, K. P., Asymmetry in the structure of the ABC transporter-binding protein complex BtuCD-BtuF. *Science* **2007**, *317* (5843), 1387-1390.
3. Tormyshev, V. M.; Chubarov, A. S.; Krumkacheva, O. A.; Trukhin, D. V.; Rogozhnikova, O. Y.; Spitsyna, A. S.; Kuzhelev, A. A.; Koval, V. V.; Fedin, M. V.; Godovikova, T. S.; Bowman, M. K.; Bagryanskaya, E. G., Methanethiosulfonate Derivative of OX063 Trityl: A Promising and Efficient Reagent for Side-Directed Spin Labeling of Proteins. *Chem. Eu. J.* **2020**, *26* (12), 2705-2712.
4. Joseph, B.; Sikora, A.; Bordignon, E.; Jeschke, G.; Cafiso, D. S.; Prisner, T. F., Distance Measurement on an Endogenous Membrane Transporter in E-coli Cells and Native Membranes Using EPR Spectroscopy. *Angew. Chem. Int. Ed.* **2015**, *54* (21), 6196-6199.
5. Sarver, J. L.; Zhang, M.; Liu, L. S.; Nyenhuis, D.; Cafiso, D. S., A Dynamic Protein-Protein Coupling between the TonB-Dependent Transporter FhuA and TonB. *Biochemistry* **2018**, *57* (6), 1045-1053.
6. Joseph, B.; Jeschke, G.; Goetz, B. A.; Locher, K. P.; Bordignon, E., Transmembrane gate movements in the type II ATP-binding cassette (ABC) importer BtuCD-F during nucleotide cycle. *J. Biol. Chem.* **2011**, *286* (47), 41008-41017.
7. Teucher, M.; Bordignon, E., Improved signal fidelity in 4-pulse DEER with Gaussian pulses. *J. Magn. Reson.* **2018**, *296*, 103-111.
8. Tait, C. E.; Stoll, S., Coherent pump pulses in Double Electron Electron Resonance spectroscopy. *Phys. Chem. Chem. Phys.* **2016**, *18* (27), 18470-18485.
9. Jeschke, G.; Chechik, V.; Ionita, P.; Godt, A.; Zimmermann, H.; Banham, J.; Timmel, C. R.; Hilger, D.; Jung, H., DeerAnalysis2006 - A Comprehensive Software Package for Analyzing Pulsed ELDOR Data. *Appl. Magn. Reson.* **2006**, *30*, pp 473-498.
10. Fabregas Ibanez, L.; Jeschke, G.; Stoll, S., DeerLab: a comprehensive software package for analyzing dipolar electron paramagnetic resonance spectroscopy data. *Magn Reson.* **2020**, *1* (2), 209-224.
11. Worswick, S. G.; Spencer, J. A.; Jeschke, G.; Kuprov, I., Deep neural network processing of DEER data. *Sci. Adv.* **2018**, *4* (8), eaat5218.
12. Polyhach, Y.; Bordignon, E.; Jeschke, G., Rotamer libraries of spin labelled cysteines for protein studies. *Phys. Chem. Chem. Phys.* **2011**, *13* (6), 2356-66.
13. Ketter, S.; Dajka, M.; Rogozhnikova, O.; Dobrynin,S. A.; Tormyshev, V. M.; Bagryanskaya, E. G.; Joseph, B., In situ distance measurements in a membrane transporter using maleimide functionalized orthogonal spin labels and 5-pulse electron-electron double resonance spectroscopy. *J Magn. Reson. Open* **2022**, *10-11*, 100041.
14. Moeck, G. S.; Letellier, L., Characterization of in vitro interactions between a truncated TonB protein from Escherichia coli and the outer membrane receptors FhuA and FepA. *J. Bacteriol.* **2001**, *183* (9), 2755-64.
15. Gopinath, A.; Joseph, B., Conformational Flexibility of the Protein Insertase BamA in the Native Asymmetric Bilayer Elucidated by ESR Spectroscopy. *Angew. Chem. Int. Ed*. **2022**, *61* (2), e202113448.
16. Ketter, S.; Gopinath, A.; Rogozhnikova, O.; Trukhin, D.; Tormyshev, V. M.; Bagryanskaya, E. G.; Joseph, B., In Situ Labeling and Distance Measurements of Membrane Proteins in E. coli Using Finland and OX063 Trityl Labels. *Chem. Eur. J.* **2021**, *27* (7), 2299-2304.
17. Joseph, B.; Jaumann, E. A.; Sikora, A.; Barth, K.; Prisner, T. F.; Cafiso, D. S., In situ observation of conformational dynamics and protein ligand-substrate interactions in outer-membrane proteins with DEER/PELDOR spectroscopy. *Nat. Protoc.* **2019,** *14* (8), 2344-2369.
